# Dissemination characteristics and influencing factors of topic circles in multi-layer public opinion dissemination

**DOI:** 10.3389/fpubh.2025.1566746

**Published:** 2025-05-07

**Authors:** Xiaoqian Zhu, Guang Yu, Yinglong Zhang, Ning Ma

**Affiliations:** School of Management, Harbin Institute of Technology, Harbin, China

**Keywords:** topic circles, dynamic network analysis, user role classification, multi-layer dissemination model, core users

## Abstract

**Introduction:**

In recent years, social media has become a pivotal channel for public opinion dissemination, characterized by its multi-topic nature and dynamic complexity.

**Methods:**

This study utilizes data on public health incident dissemination from the Weibo platform as its research sample and, based on a multi-layered network model, proposes a framework for topic circle identification and dynamic network analysis in public opinion dissemination. The study explores the classification of user roles within topic circles, constructs a dynamic role transition matrix, and quantitatively analyses the impact of topic circle characteristics on dissemination volume.

**Results:**

The results indicate that core users exhibit significant stability and a dominant role within dissemination networks, while intermediate users display pronounced role mobility, and peripheral users are most sensitive to topic relevance. Furthermore, sentiment, forwarding enthusiasm, and forwarding depth exhibit significant differences in their effects on dissemination volume across different user roles.

**Discussion:**

This study enriches public opinion dissemination theory by examining the dynamic evolution of topics and user role transitions, providing practical guidance for managing and controlling public opinion dissemination on social media.

## 1 Introduction

In recent years, social media has become a key channel for the dissemination of public opinion in response to public events, with its dissemination characteristics exhibiting multi-topic diversity, emotional variability, and dynamic complexity (1). Weibo, one of the most representative social media platforms in China (2), not only facilitates large-scale information interactions but also drives multi-layered diffusion of public opinion through its unique forwarding network. In this virtual social sphere of social media, user engagement is characterized by a “circle” structure (3), whereby people with similar interests in a particular topic group together to form “circles.”

However, the influence mechanism of topic evolution and relatedness on user behavior during the dynamic process of public opinion dissemination has yet to be fully explored. Existing studies on social media public opinion mainly focus on the impact of user characteristics (such as gender, number of followers, and sentiments) and content features on dissemination effect (4–6). However, there are still some gaps: First, there is a lack of systematic analysis on the dynamic evolution and relatedness of public opinion topics; second, there has been insufficient in-depth study on the dissemination behaviors of different user roles within dynamic networks; third, current methods struggle to quantify the role of multi-layered interactions among users in diffusion processes.

To address these issues, this study selects a widely discussed public health incident - the “Big-Headed Baby” case caused using antibacterial cream - as a research case. The event originated on 7 January 2021, when a well-known Weibo blogger, “Dad's Review - Wei Laoba,” posted a video on the Weibo platform stating that “a baby in Fujian turned into a big-headed baby after using antibacterial cream.” This incident sparked extensive discussion, with multiple Weibo topics such as “#Baby Turns Big-Headed After Using Antibacterial Cream#” and “#Hormonal Baby Cream#” rapidly going viral and attracting tens of thousands of participants. This incident serves as a typical case for studying public opinion dissemination on social media.

Given the complexity and multi-layered nature of public opinion dissemination on social media, this study aims to conduct an in-depth investigation into the dissemination network of public opinion incidents and the thematic evolution characteristics of its multi-layered structural in the context of mass communication on social media. To this end, this study introduces the novel concept of 'topic circles' to characterize the dynamic features of thematic evolution in public opinion dissemination networks. By integrating dynamic network coreness analysis, this study seeks to uncover the interaction mechanisms between user behavior and topic characteristics, thereby providing an effective theoretical approach for managing the increasingly complex dissemination of public opinion in social networks. The study aims to address the following research questions: (1) Does public opinion dissemination on social media exhibit a multi-layered dissemination network model? (2) Is the multi-layered dissemination network composed of distinct topic circles? (3) What are the role types of users participating in dissemination within circles, and how do they influence dissemination? (4) What are the key factors influencing the effectiveness of multi-layered public opinion dissemination?

To address these questions, this study constructs a multi-layered public opinion dissemination network analysis framework based on Weibo platform data. Through topic classification, coreness analysis, and other methods, it systematically quantifies the complex relationship between topic circle dissemination characteristics and user behaviors, providing more effective theoretical approaches for managing social media public opinion. The remainder of this paper is structured as follows: Section 2 reviews relevant research; Section 3 presents the theoretical framework and research methods; Section 4 conducts empirical research and result analysis based on public opinion events; and, finally, management recommendations are provided based on the research findings.

## 2 Literature review

Social media has become a crucial platform for public opinion dissemination, with its dissemination characteristics typically marked by rapid diffusion, multi-layered dissemination structures, and significant impacts from both the content and sentiment on dissemination behavior (4). Research has shown that, in the layered dissemination model of public opinion, the evolution of topics across different layers exhibits distinct dynamic characteristics (7). For instance, methods such as Latent Dirichlet Allocation (LDA) and Bidirectional Encoder Representations from Transformers (BERT) have shown remarkable effectiveness in identifying key topics in the dissemination of public opinion (8–10). Although traditional methods can effectively analyse events in stages, revealing the topic characteristics at each stage, they often fail to systematically capture the connections between different time periods (7) and fail to fully explore how dissemination paths affect the dynamic evolution of topics (11). Additionally, some scholars have examined user behavior characteristics within the dissemination chain of public opinion topics (8).

Secondly, the role of user roles in public opinion dissemination has garnered significant attention. Some studies have employed algorithms such as PageRank or core-periphery analysis (12–14) to identify the pivotal roles that users play in public opinion networks. Chen et al. (15) revealed the different roles played by the general public, opinion leaders, and organizations in the dynamic evolution of COVID-19-related topics, illustrating the differences in information dissemination and influence across these groups. Wang et al. (16) proposed a multi-dimensional similarity-based classification and identification algorithm using K-shell decomposition, effectively identifying key users in the dissemination of public opinion topics, such as opinion leaders, focus figure, and communication figure, and revealing their roles in different stages of dissemination. D. Wang et al. (17) divided key users into opinion leaders and structural hole spanners, finding that common users and retweeters acted as “bridges” in the dissemination of different topics. Research shows that the role of different users in public opinion dissemination is dynamic, with bridge users, in particular, becoming more prominent in cross-domain dissemination; the complex structure of public opinion dissemination networks and roles also evolves dynamically (11).

Furthermore, the driving factors of public opinion dissemination are often characterized by the interactive effects of multiple variables, including topic and sentiment relevance, forwarding depth, and forwarding enthusiasm (18–21). The role of sentiment in public opinion dissemination has received considerable attention in recent years. Relevant studies have indicated that positive and negative sentiment have significantly different effects on the breadth and depth of information dissemination. For instance, Luo et al. (4) demonstrated through their study of COVID-19 public opinion data that negative sentiment significantly drive the depth and breadth of information dissemination, while positive sentiment tend to stimulate the sustained involvement of core users. Sun et al. (14) emphasized that negative sentiment have a more pronounced impact on short-term user dissemination behavior, particularly during the early stages of the diffusion of internet hotspots. Moreover, forwarding depth and forwarding enthusiasm are crucial dimensions for measuring dissemination behavior. Li et al. (22) found that in the social media dissemination following natural disasters, posts related to anger significantly increased reposting scale, while posts containing anxious emotions from highly followed users inhibited dissemination. Meng et al. (23) revealed that anger facilitated the depth of dissemination, sadness helped increase dissemination breadth, and disgust enhanced both dissemination depth and structural dissemination.

Despite significant progress in topic dynamic analysis, sentiment contagion, and user role identification in social media public opinion dissemination, existing research still faces several limitations as the widespread application of new social media technologies makes public opinion dissemination increasingly complex. First, traditional topic modeling methods struggle to effectively capture the dynamic evolution of topics and their impact on dissemination structure and effects. Second, user role classification methods typically rely on static analysis, lacking an in-depth exploration of role evolution and the interactions between topics. Finally, research on sentiment contagion has yet to sufficiently consider the layered effects in multi-role scenarios.

To address the shortcomings of previous studies, this paper introduces the concept of “topic circles” based on the multi-layered network structure of public opinion dissemination. By categorizing dissemination paths according to topic characteristics and integrating dynamic network analysis techniques, it reveals the interactive nature of topic evolution and dissemination pathways. At the same time, considering the dynamic changes in user roles, the paper uncovers the sentiment differentiation across various role groups. Through the quantification of the interaction effects between sentiment, topic relevance, and reposting behavior, this study reveals the complex relationships between user roles, sentiment transmission, and dynamic network characteristics. In doing so, it provides a more comprehensive dynamic analysis framework for understanding public opinion dissemination patterns and offers new theoretical insights for sentiment-based intervention strategies targeting different user groups in public crisis management.

## 3 Research methods

**Figure 1:**
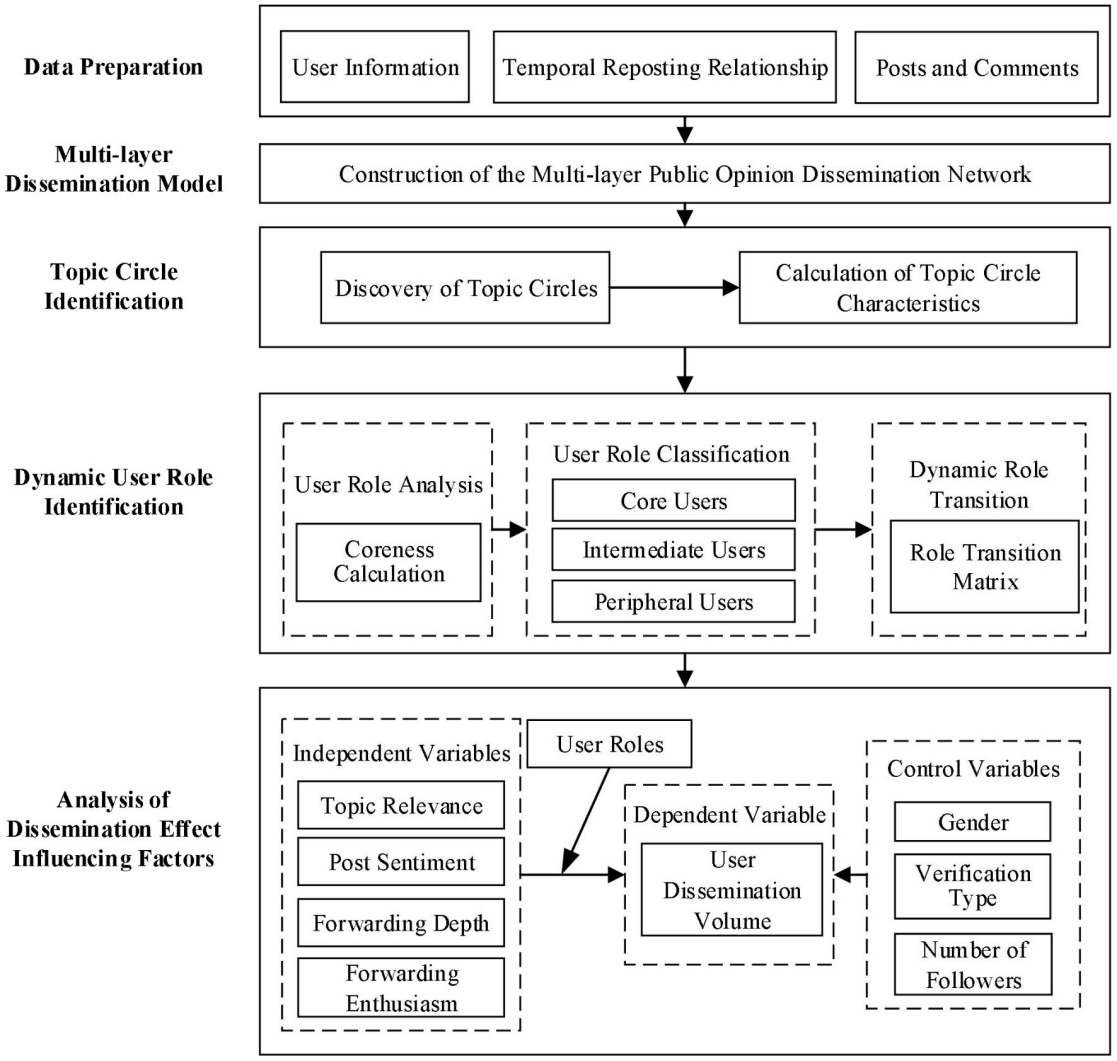
Technical roadmap.

This study encompasses four main aspects. First, a multi-layered network model for public opinion dissemination is constructed using public opinion dissemination events as case studies. Second, the main topics of public opinion events in each circle are identified, and topic circles are constructed using user reposting relationships. Third, dynamic network analysis methods are used to calculate user coreness within topic circles. Users are then classified into roles through clustering algorithms, and a role transition matrix is constructed to analyse their dynamic evolution. Finally, a negative binomial regression model is employed to investigate the impact of key factors, including user roles, topic relevance, sentiment, and forwarding depth, on the propagation scale of topic circles. The research design and technical roadmap are shown in Figure 1.

### 3.1 Data collection and preprocessing

The data collection period spanned from 6 January 2021 to 6 February 2021. Using the keywords “antibacterial cream” and “big-headed baby,” 121,534 Weibo posts related to the event were collected through web scraping techniques. These posts included 2,063 original posts and 119,471 reposts. In addition, relevant user information such as gender, account registration date, account verification, and follower count were collected, along with dissemination metrics for each post (e.g., number of reposts and comments). To ensure data quality, irrelevant posts (e.g., unrelated content, advertisements, and duplicate entries) were manually removed as noise data, resulting in 84,844 valid entries for subsequent analysis.

During data collection, a Weibo data extraction tool based on API access was employed. To ensure compliance in data collection, all user data were anonymised during the analysis process. Specifically, all user identifiers (e.g., usernames and account IDs) were removed to prevent the exposure or identification of personal information. The dataset includes only publicly available posts and user information, excluding private or restricted-access data.

Furthermore, this study strictly adheres to social media platform policies and relevant legal regulations, fully respecting user privacy and data security throughout the data collection and usage process. All collected data are exclusively used for academic research and will not be utilized for any commercial purposes or dissemination. The data processing procedure strictly follows ethical review protocols and institutional regulations, ensuring full compliance with ethical standards.

### 3.2 Construction of the multi-layer public opinion dissemination network

To investigate the structural and evolutionary characteristics of public opinion dissemination events, a multi-layered public opinion dissemination network was constructed. Social media users were defined as nodes, and reposting behaviors as directed edges, with the direction of the edge pointing from the reposted user to the re-poster, reflecting the pathways of information dissemination.

First, LDA model was applied to identify topics within each circle, segmenting dissemination content into distinct “topic circles.” For each topic, core content and a preliminary set of users associated with the topic were identified, providing the basis for extracting the dissemination network.

Next, based on reposting data from dissemination events, multi-level reposting relationships and temporal information among “topic circle” users were extracted. A dissemination network centered on user reposting behaviors was constructed for each topic. The data was then sliced into 12-h windows using timestamps to create a time-series dynamic dissemination network.

Finally, duplicate UIDs were removed, and link completion was performed to generate a complete dissemination network. Isolated nodes were excluded, and the network data was converted into formats compatible with tools such as Pajek and Gephi for visualizing the network structure and dissemination characteristics. Results showed that the degree distribution of the network followed a power-law distribution, consistent with the typical characteristics of social networks.

### 3.3 Topic circle extraction and feature analysis

#### 3.3.1 Topic extraction

Topic extraction serves as the initial step of this research, aiming to identify the main topics from large-scale social media data to reveal opinion leaders and dissemination mechanisms within each topic. LDA was used as the primary method for topic identification, uncovering latent topics from original and reposted posts in public opinion events and quantifying the text distribution under each topic.

LDA, as a probabilistic topic modeling approach, is highly scalable and capable of unsupervised learning, making it suitable for processing the complex and fragmented textual content found in social media. It effectively handles large volumes of text data and uncovers latent topics and their distributional characteristics (7). The LDA model was implemented with the following hyperparameter configurations:

- Number of topics (num_topics): 6
- Number of passes: 20
- Iterations per pass: 100
- Alpha: ‘auto' (automatic adjustment of document-topic distribution sparsity)
- Eta: 0.01 (controlling topic-word distribution sparsity)
- Random seed: 42

These parameter configurations were optimized to ensure efficient topic extraction while maintaining model reproducibility. The LDA model was implemented using the Gensim library in Python.

For sentiment analysis, the study utilized the BERT model, which was built and trained using the TensorFlow deep learning framework. Data processing was conducted with Pandas, while the implementation and support for the BERT model are provided by the Transformers library from Hugging Face, ultimately enabling the completion of the sentiment classification task in Python.

#### 3.3.2 Characterization of topic circles

The characteristics of topic circles are described across three key dimensions: dissemination breadth, depth, and speed, which can be represented as a multi-dimensional vector:


$$\begin{array}{l} V=\left[S_{r},S_{u},D_{\max},D_{avg},V,E\right] \end{array}$$

Where *S* represents the dissemination scale, comprising user scale *S*_*u*_ and repost relationship scale *S*_*r*_; *D*_max_ denotes the maximum forwarding depth, reflecting the extent of topic engagement and interaction activity; *D*_*avg*_ represents the average forwarding depth, used to measure the hierarchical nature of information diffusion; *V* indicates the dissemination speed, which serves as a proxy for diffusion efficiency; and *E* refers to the average sentiment value, offering insights into the sentiment dynamics of dissemination.

**Figure 2:**
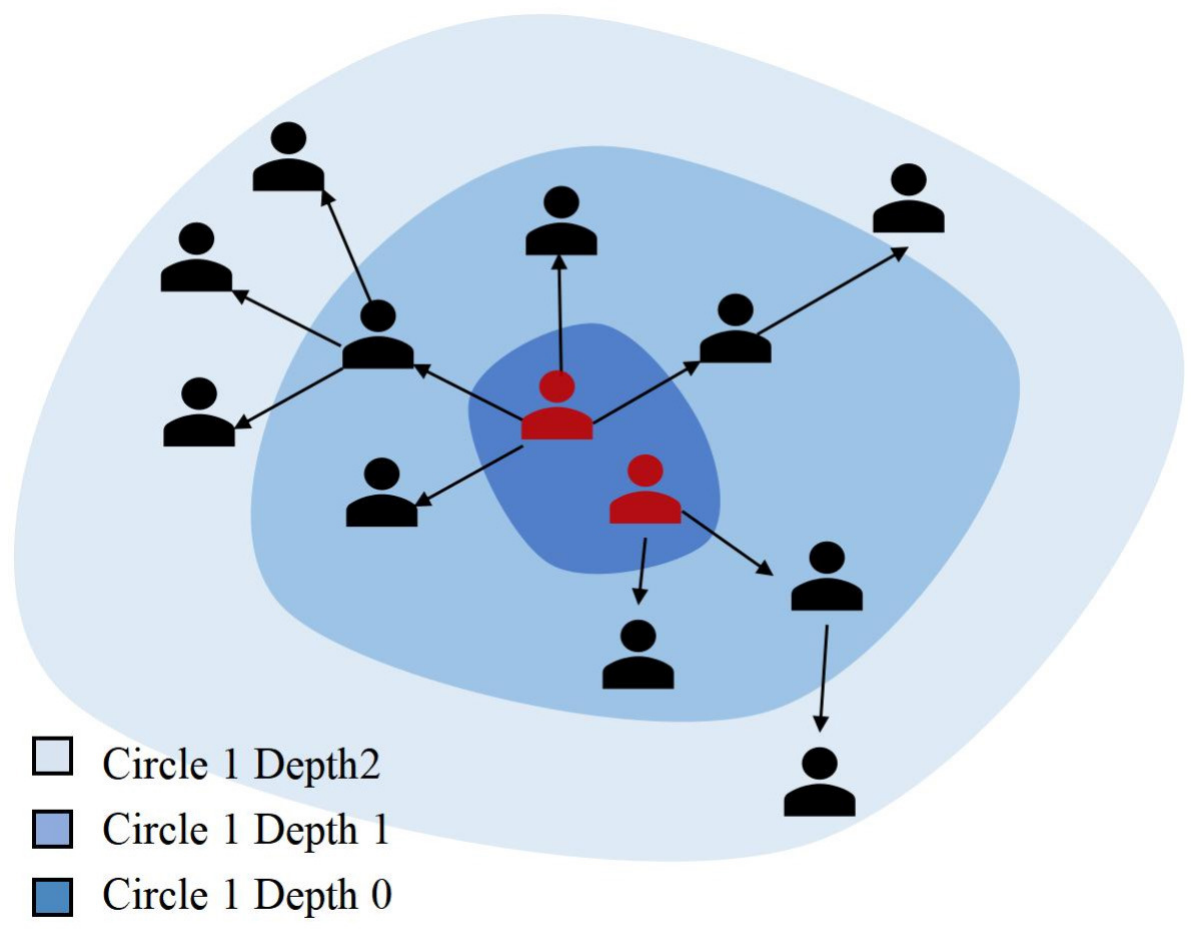
Schematic diagram of topic circles and forwarding depth.

A schematic diagram of topic circles and their forwarding depth is shown in Figure 2. Red nodes represent original users, while black nodes represent reposting users. The dark blue area corresponds to depth 0 of Circle 1, and the light blue area represents depth 2 of Circle 1. Assuming all users repost only once, Circle 1′s maximum dissemination depth is 2, with a user scale of 12 and a reposting relationship scale of 11.

### 3.4 Dynamic identification of user roles

#### 3.4.1 Calculation of user coreness in topic circles

To quantify users' dissemination capabilities in the multi-layered public opinion network, the Hubs score from the HITS algorithm is employed to calculate user coreness, evaluating users' influence as sources of information diffusion.

#### 3.4.2 User role classification and dynamic transition analysis

Based on the distribution of user coreness within each time window, users were classified into three categories using the K-means clustering algorithm: core users, intermediate users, and peripheral users. Core users occupy a dominant position in the dissemination network, intermediate users serve as bridges connecting network nodes, and peripheral users exhibit the lowest centrality, typically functioning as information receivers or low-participation nodes at the network's periphery.

To further analyse the dynamic changes in user roles, a role transition matrix was constructed to quantify the mobility of different roles between adjacent time windows. The process involved the following steps: First, initialize a 3 x 3 matrix to represent the transition relationships between the three roles (core users, intermediate users, and peripheral users). Second, compare each node's role in adjacent time windows and counting the number of transitions between roles. Finally, normalize the transition matrix to obtain a transition probability matrix. The transition probability matrix reflects the temporal dynamics of node roles, capturing their evolution over time.

#### 3.4.3 Dynamic role classification method

Dynamic role classification aims to determine the dominant role of nodes throughout the study period via time-series analysis. First, the proportion of time each node was classified as a core role, intermediate role, or peripheral role across all time windows was calculated. Next, the role with the highest proportion was defined as the node's dynamic role. For example, if a node exhibited the highest proportion in the core role category, its dynamic role was identified as a core role. To ensure reproducibility of the analysis, data on the role evolution of each node was consolidated and archived, including detailed information on role changes across all time windows.

#### 3.4.4 Demographic analysis of core, intermediate, and peripheral users

To further understand the demographic differences among core, intermediate, and peripheral users, this study conducts a grouped statistical analysis of user gender, log-transformed number of followers, log-transformed number of microblogs, log-transformed number of followings, registration time, and verification type. The Demographic statistics of user roles are shown in Table 1.

**Table 1:** Demographic statistics of core, intermediate, and peripheral users.

**Variable**	**Core users M (SD)**	**Intermediate users M (SD)**	**Peripheral users M (SD)**
Gender	0.32 (0.46)	0.31 (0.46)	0.24 (0.43)
Number of followers (ln)	2.22 (1.27)	3.02 (1.21)	2.15 (0.85)
Number of microblogs (ln)	6.72 (2.15)	8.71 (1.82)	7.24 (1.93)
Number of followings (ln)	5.66 (1.20)	6.20 (0.97)	5.77 (1.01)
Authentication	0.10 (0.30)	0.21 (0.41)	0.05 (0.22)
Registration year	2014.14 (3.09)	2012.85 (3.00)	2013.76 (3.00)
*N*	1,951	3,772	70,065

Values represent M (SD) unless otherwise noted. Gender: 0 = female, 1 = male; Authentication: 0 = ordinary user, 1 = celebrity; ln = natural logarithm transformation.

The results indicate that across all user roles, the proportion of female users is significantly higher than that of male users, particularly among peripheral users, where females account for as much as 76%. Moreover, the average log-transformed number of followers of intermediate users (3.02) is significantly higher than that of core users (2.22) and peripheral users (2.15). Additionally, intermediate users exhibit a significantly higher log-transformed number of microblogs (8.71) and log-transformed number of followings (6.20) compared to other user roles.

These findings suggest significant demographic differences among different user roles, which may be related to their roles and behaviors within the dissemination network.

The above analysis indicates significant demographic differences among core, intermediate, and peripheral users. These differences may influence their roles and behaviors within the dissemination network, thereby affecting the dissemination scale of topic circles. Next, a negative binomial regression model is employed to further examine the impact of user roles, topic relevance, sentiment, and forwarding depth on the dissemination scale of topic circles.

### 3.5 Factors influencing the dissemination effectiveness of topic circles

#### 3.5.1 Measures of variables

##### 3.5.1.1 Dependent variable

The dependent variable primarily measures the dissemination influence of public opinion, defined as dissemination volume. This variable serves as an indicator of a user's direct influence within the public opinion dissemination network, specifically reflected by the number of reposts their posts receive. By analyzing the reposting relationships in the dynamic network, the direct reposting volume for each user is calculated to reflect the strength of their dissemination influence. Theoretically, reposting serves as a critical driving factor in information dissemination (21).

##### 3.5.1.2 Independent variables

The independent variables primarily include the attribute characteristics of topic circles, detailed as follows:

User roles measure the functional positioning of users within the dissemination network, classified into three categories: core users, intermediate users, and peripheral users (24). Based on user behavioral data in the dynamic network, the K-means clustering algorithm is employed to categorize users according to their coreness. Core users represent the most influential nodes in dissemination, intermediate users serve as bridges connecting network nodes, and peripheral users exhibit weaker dissemination capabilities. Role classification reveals the functional characteristics of users within the dissemination network, supporting the analysis of dissemination structures (16).

Topic relevance measures the degree of content similarity between a user's post and the source post, reflecting the user's contribution to content dissemination. Using the TF-IDF model, all post content is vectorised, and the cosine similarity between each post's text vector and the source post's vector is calculated. Topic relevance captures content consistency during the dissemination process and the evolutionary patterns of public opinion (17).

Post sentiment is classified into three types: positive, neutral, and negative. Sentiment analysis is conducted using BERT model, which overcomes the limitations of informal language in social media texts and efficiently captures contextual information (25). A random sample of 31,000 posts was manually annotated, achieving a Cohen's Kappa coefficient of 0.710 to validate annotation consistency. The dataset was split into training and testing sets at a ratio of 8:2, with balanced sentiment label distribution (1:1:1). The model achieved a classification accuracy of 83.62% on the test set.

Forwarding depth is defined as the shortest path length between a node and the source node, calculated based on the hierarchical structure of the dynamic network. It reflects the diffusion range and hierarchical characteristics of information within the network (23). Forwarding enthusiasm measures the responsiveness of users to dissemination events, determined by normalizing the time interval between a user's post and the source post. Higher forwarding enthusiasm indicates quicker user responses and greater proactivity in engaging with events (26).

##### 3.5.1.3 Control variables

To eliminate the potential interference of user attributes on dissemination influence and enhance model accuracy, user attributes were included as control variables. These attributes comprise gender, number of followers, and verification type. Gender was categorized as male or female. The number of followers reflects a user's popularity, while verification type distinguishes between ordinary users and celebrity groups, capturing differences in user identity attributes.

### 3.5.2 Selection of regression method

Negative binomial regression was employed in this study for analysis. This method effectively addresses the issue of overdispersion and allows the use of count variables as dependent variables. As shown in Table 2, the mean of dissemination volume is significantly smaller than its variance, confirming the presence of overdispersion in the variable. Thus, the negative binomial regression model is more appropriate for explaining the driving factors of this variable. The descriptive statistics of the variables are shown in Table 2.

**Table 2:** Descriptive statistics of variables.

**Variables**	**Description**	**Mean (SD)**
**Dependent variable**		
Dissemination Volume	User dissemination volume within topic circles.	5.65 (246.40)
**Independent variables**		
User Roles	Core users, intermediate users, and peripheral users.	2.87 (0.40)
Topic Relevance	Topic relevance.	0.68 (0.29)
Forwarding Depth	The hierarchical position of users within the dissemination network.	1.91 (1.34)
Positive Sentiment	Binary variable: 0 or 1.	0.03 (0.16)
Negative Sentiment	Binary variable: 0 or 1.	0.25 (0.43)
Reposting Proactivity	User responsiveness to dissemination events, ranging between [0, 1].	25.92 (48.47)
**Control variables**		
Genders	1 for male, 0 for female.	0.25 (0.43)
Authentication	Verification type: 0 for ordinary users, 1 for celebrities.	0.08 (0.27)
Ln_Followers	Natural logarithm of the user's follower count.	2.26 (0.96)

## 4 Results analysis

### 4.1 Topic circle discovery based on topic identification

#### 4.1.1 Topic identification

The content of original posts and reposts was analyzed using the LDA model. By adjusting model parameters, the optimal number of topics was determined to be 6 based on perplexity and coherence score metrics. These topics achieved the best performance with a perplexity score of −6.941 and a coherence score of 0.5401. Each topic is represented by a set of high-frequency keywords, reflecting the primary concerns of the public during the dissemination of public opinion. Detailed results are shown in Table 3.

**Table 3:** Topic categories.

**No**.	**Proportion**	**Topic**	**LDA keywords (Top 10)**
1	43.38%	Product Quality and Hormone Controversy	big-headed, infant, Dad, post-cream, Fujian, Zhangzhou, parents, special care, child, growth
2	23.93%	Parental Concerns about Eczema Treatment	hormone, heartache, pomelo, baby, eczema, neck, topical use, long-term, guardian, moisturizer
3	16.80%	Special Care Products in Fujian Context	antibacterial, involved, Fujian, enterprise, testing, manufacturing, additive, reporter, recall, wellbeing
4	11.77%	Public Discourse on Baby Products	baby, guardian, involved, post-cream, hype, blogger, response, social media, exposure, incident
5	2.35%	Corporate Responsibility and Regulation	product, involved, baby, inspection, production, antibacterial, concentration, company, manufacturer, withdrawal
6	1.77%	Hormonal Side Effects in Eczema Treatment	infant, hormone, doctor, eczema, treatment, Dad, side effects, cream, ointment, review

To enhance the readability and appeal of this study, representative posts were selected from each topic to illustrate specific content and key public concerns in public opinion dissemination. Detailed results are shown in Table 4.

**Table 4:** Topic analysis of public opinion on Weibo.

**Topic no**.	**Topic description**	**Representative posts**	**User ID**	**Post date**
1	Product Quality and Hormone Controversy	“My baby developed redness and peeling after using this antibacterial cream. It's terrifying! I hope the authorities will strictly investigate these substandard products!”	#001	2021-01-07
		“This cream contains hormones! Long-term use is extremely harmful to babies. Parents must be vigilant and not be deceived by merchants!”	#002	2021-01-08
2	Parental Concerns about Eczema Treatment	“My baby also has eczema. The doctor recommended hormonal ointment, but after this news, I'm really conflicted about whether to use it.”	#003	2021-01-10
		“As a parent, it's heartbreaking to see babies suffering from substandard products. I hope the authorities will strengthen supervision to protect our children!”	#004	2021-01-12
3	Special Care Products in Fujian Context	“The involved company is from Fujian. I heard local investigations have begun. Hope they release results soon to give parents answers!”	#005	2021-01-09
		“Fujian companies producing such substandard products is shameful! Hope the authorities severely punish these unethical businesses!”	#006	2021-01-12
4	Public Discourse on Baby Products	“Is this event being hyped? Why is everyone suddenly discussing it?”	#007	2021-01-08
		“Physical stores should have social responsibility. With such high rents, they should be more careful about what they sell.”	#008	2021-01-10
5	Corporate Responsibility and Regulation	“Shouldn't companies be held accountable for producing substandard products? Hope the authorities investigate thoroughly!”	#009	2021-01-13
		“Regulators should strengthen supervision and random checks on baby care products to prevent substandard products from entering the market.”	#010	2021-01-13
6	Hormonal Side Effects in Eczema Treatment	“My baby also has eczema. The doctor prescribed hormonal ointment, but after this news, I'm worried about long-term side effects.”	#011	2021-01-17
		“While hormonal ointments can relieve eczema, long-term use does carry risks. Parents should be cautious and follow medical advice.”	#012	2021-01-18

Data collected from Weibo platform between 2021-01-01 and 2021-02-06. User IDs have been anonymised to protect privacy. Posts have been translated from Chinese while maintaining original meaning.

The above posts illustrate the typical discussion content within each topic, reflecting public concerns regarding the quality of infant care products, corporate responsibility, and regulatory issues. Next, a further analysis of the temporal evolution patterns of each topic will be conducted to examine the variations in topic popularity during public opinion dissemination.

#### 4.1.2 Analysis of temporal trends in topic popularity

**Figure 3:**
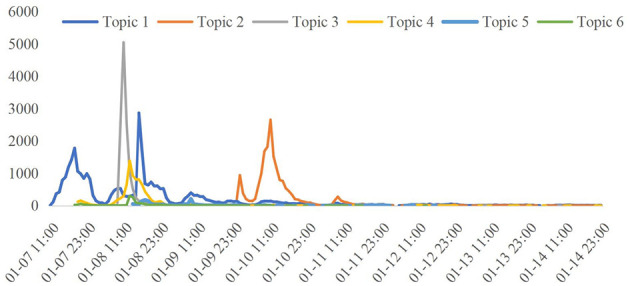
Trends in topic popularity.

We further analyzed the temporal evolution patterns of each topic (as shown in Figure 3). The changes in topic popularity reflect the contribution and influence of each topic on public opinion dissemination across different time periods.

Figure 3 illustrates the popularity trends of six major topics during the evolution of public opinion. Topic 1 (43.38%) reached its peak in the early stages of the event (7–8 January), with public discussions focusing primarily on product safety and debates over hormonal content, before rapidly declining in popularity. Topic 2 (23.93%) peaked between 9–12 January, driven by widespread emotional resonance sparked by parents' voices. Topic 3 (16.80%) saw heightened popularity from 9–13 January as public attention shifted to the company's background and product authenticity. Topic 4 (11.77%) exhibited relatively stable popularity, reflecting ongoing interest in social media hype and the company's responses. Topic 5 (2.35%) emerged after 13 January, with discussions transitioning from the specific event to broader topics such as policies, regulations, and corporate accountability. Although its popularity was low, it persisted over a longer period. Topic 6 (1.77%), the least popular topic, appeared briefly in the later stages, mainly covering scientific explanations and educational content.

Overall, the trajectory shows that Topic 1 dominated the initial public opinion peak, followed by Topics 2 and 3 driving discussions in the mid-phase. In the later stages, Topics 5 and 6 became the focus of long-tail interest.

#### 4.1.3 Analysis of topic circle characteristics

Table 5 analyses the dissemination characteristics of different topic circles across three dimensions: dissemination scale, dissemination depth, and dissemination speed. Additionally, it incorporates average sentiment data to provide insights into the emotional dynamics within each circle.

**Table 5:** Characteristics of topic circles.

**Topic circle**	**Dissemination scale**		**Dissemination depth**		**Dissemination speed**	**Average sentiment**
	**Reposts**	**Users**	**Max depth**	**Avg depth**	**Avg speed**	
1	36,805	33,623	23	2.23	51.05	−0.28
2	20,303	18,795	19	1.87	48.63	−0.08
3	14,254	13,119	5	1.13	33.83	−0.30
4	9,986	8,752	14	1.99	21.19	−0.23
5	1,994	1,351	4	1.44	6.36	−0.12
6	1,502	1,387	9	1.60	3.95	−0.02

In terms of dissemination scale, Circle 1 exhibits the strongest performance across all dimensions, with a user scale of 33,623, a repost relationship scale of 36,805, a maximum dissemination depth of 23, and an average dissemination speed of 51.05 posts per hour. These metrics indicate exceptionally high coverage and activity levels. In contrast, Circle 6 demonstrates the most limited dissemination, with a user scale of only 1,387 and a dissemination speed of 3.95 posts per hour, suggesting that discussions around this topic are narrow in scope and primarily concentrated within highly specialized groups.

Dissemination depth reveals the hierarchical structure of information diffusion. Circle 1′s average dissemination depth of 2.23 and maximum depth of 23 reflect heightened public interest in issues related to the quality and hormonal safety of infant products. Circle 2 also demonstrates considerable dissemination depth, suggesting that comments from parents of infants are particularly effective in evoking resonance.

In terms of emotional analysis, Circles 3, 1, and 4 exhibit higher levels of negative sentiment, reflecting public outrage over quality issues with infant care products. These circles primarily focus on concerns related to product quality.

Overall, Circle 1 stands out with the strongest performance in dissemination breadth, depth, and speed, indicating the highest levels of public attention and opinion risk. In contrast, Circle 6 shows the lowest dissemination range and speed, coupled with neutral sentiment, indicating that discussions within this circle are more specialized and professional. This analysis provides a multi-dimensional perspective for understanding the dissemination patterns of different topics. By uncovering the heterogeneity in dissemination mechanisms across circles, it offers valuable data to support public opinion management and risk assessment.

### 4.2 Dynamic evolution analysis of user roles

#### 4.2.1 Role transition matrix analysis

**Figure 4:**
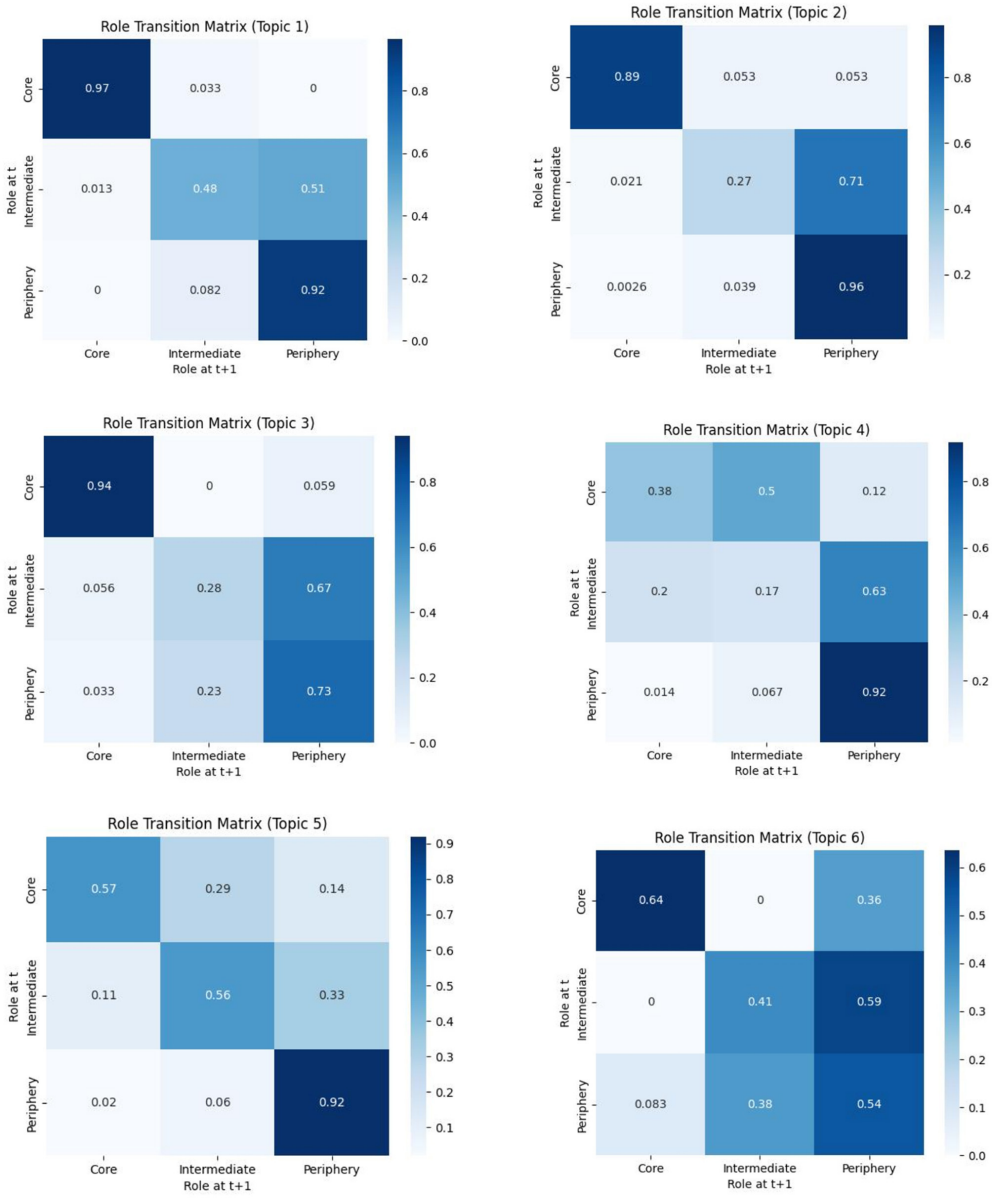
Role transition matrix.

Based on topic circles, users were classified into core users, intermediate users, and peripheral users. A normalized role transition matrix was constructed to quantify role mobility between adjacent time windows. Detailed results are shown in Figure 4. The analysis reveals that core user are relatively stable. For instance, in Topic Circle 1, the probability of core users maintaining their core role is 97%. However, some core users may transition to intermediate or peripheral roles, as observed in Circles 4, 5, and 6.

Intermediate users exhibit high mobility, with the potential to either ascend to core roles or descend to peripheral roles. In Circle 4, the probability of intermediate users transitioning to peripheral roles is as high as 63%, while the probability of being promoted to core roles is 20%. This bidirectional mobility underscores the bridging role of intermediate users within the dissemination network.

Peripheral users show minimal role changes, with the majority maintaining their original role. For example, in Circle 2, the probability of peripheral users remaining in their role is 96%. However, a small number of active peripheral users may ascend to intermediate or core roles.

In summary, core users exhibit the highest role stability, intermediate users demonstrate the greatest mobility, and peripheral users undergo the least change. The role transition matrix provides robust support for the quantitative analysis of user role evolution.

#### 4.2.2 Analysis of user roles

**Figure 5:**
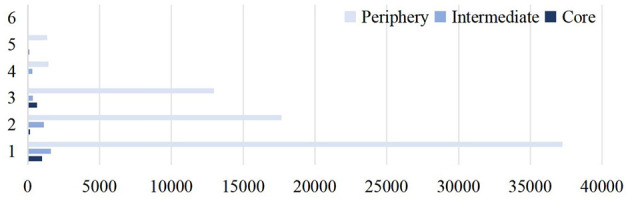
Proportion of user roles.

Using time-series analysis, the dominant roles of users within each topic circle over the period were identified. Detailed results are shown in Figure 5. The results reveal significant differences in role distribution among user groups. Peripheral users constitute the largest group, with ~70,000 participants. While they engage widely, their dissemination capability is limited. Intermediate users, numbering 3,772 (4.98%), act as bridges in the diffusion of information, facilitating connections within the network. Core users, though the smallest group at 1,951 (2.57%), possess the strongest dissemination capability and serve as the critical nodes for information diffusion.

Overall, core users dominate the dissemination process, intermediate users provide connectivity for information flow, and peripheral users contribute to the scale and reach of the network.

### 4.3 Regression results

This study employs a negative binomial regression model to analyze the factors influencing users' dissemination volume. The overall model's pseudo-*R*^2^ is 0.454, indicating that the selected variables explain 45.4% of the variation in dissemination volume. The results show significant differences between core users and peripheral users in their roles within the dissemination process. Furthermore, topic relevance, forwarding Enthusiasm, forwarding depth, and sentiment all have a significant impact on dissemination volume.

#### 4.3.1 User role differences

The regression results are shown in column (1) of Table 6. The analysis reveals significant differences in user roles within information dissemination. Core users exhibit substantially higher dissemination volumes compared to intermediate users (coefficient 3.893, *p* < 0.01), highlighting their dominant position and powerful amplification effect in the diffusion process. In contrast, peripheral users have significantly lower dissemination volumes than intermediate users (coefficient −3.622, *p* < 0.01), indicating their lower level of participation and influence. Core users play a crucial role in the network, and the diffusion of information heavily relies on their engagement, whereas peripheral users primarily act as information receivers. Thus, prioritizing the mobilization of core users is a key strategy for enhancing dissemination effectiveness, while the dissemination behaviors of peripheral users are more influenced by contextual factors and content.

**Table 6:** Negative binomial regression results.

	**(1)**	**(2)**	**(3)**	**(4)**
**Variables**	**Dissemination Volume**	**Dissemination Volume of core users**	**Dissemination volume of intermediate users**	**Dissemination volume of peripheral users**
Core user	3.893^***^			
	(9.20)			
Peripheral user	−3.622^***^			
	(−50.88)			
Topic relevance	1.299^***^	1.407^***^	1.040^***^	2.851^***^
	(10.26)	(3.82)	(13.18)	(17.79)
Forwarding enthusiasm	2.014^***^	8.715^***^	1.045^***^	4.201^***^
	(7.41)	(8.46)	(5.51)	(11.40)
Forwarding depth	0.151^***^	0.393^***^	0.100^***^	0.249^***^
	(7.71)	(4.99)	(8.08)	(9.67)
Positive sentiment	−0.105	4.306^***^	−0.344^**^	0.283
	(−0.45)	(13.08)	(−2.17)	(1.10)
Negative sentiment	0.272^***^	0.472^*^	0.093^*^	0.383^***^
	(3.73)	(1.84)	(1.85)	(5.17)
Gender	0.183^**^	0.513^**^	−0.117^**^	0.557^***^
	(2.26)	(2.21)	(−2.25)	(7.02)
Ln_Followers	1.411^***^	1.429^***^	0.876^***^	1.990^***^
	(27.39)	(13.41)	(23.12)	(30.28)
Authentication	0.028	0.551	−0.265^***^	0.165
	(0.18)	(1.58)	(−3.28)	(1.06)
Constant	−5.693^***^	−9.361^***^	−3.287^***^	−12.231^***^
	(−29.06)	(−17.80)	(−30.25)	(−49.49)
Observations	75,788	1,951	3,772	70,065
Pseudo R-squared	0.454	0.308	0.177	0.256

Robust z-statistics in parentheses.

^***^p < 0.01, ^**^p < 0.05, ^*^p < 0.1.

#### 4.3.2 Factors influencing dissemination effectiveness

The regression results are presented in columns (2), (3), and (4) of Table 6. The specific manifestations of the dissemination impact factors as follows:

Firstly, topic relevance has a significant positive effect on the dissemination volume across all user groups, with the strongest impact on peripheral users (coefficient 2.851, *p* < 0.01). This suggests that a high degree of relevance between the post content and the core event has the greatest influence on the dissemination volume of peripheral users. Core users (coefficient 1.407, *p* < 0.01) and intermediate users (coefficient 1.040, *p* < 0.01) are also significantly influenced by topic relevance, although the effect is comparatively weaker. Secondly, forwarding enthusiasm has a significant positive impact on dissemination volume, with the most pronounced effect on core users (coefficient 8.715, *p* < 0.01), indicating that their rapid response and active retweeting significantly enhance the dissemination effectiveness. Moreover, forwarding depth also has a positive effect on dissemination volume across all user groups, although the depth effect is noticeably stronger for core users (coefficient 0.393, *p* < 0.01) compared to intermediate users (coefficient 0.100, *p* < 0.01) and peripheral users (coefficient 0.249, *p* < 0.01).

The impact of sentiment varies across user groups. Positive sentiment significantly promote dissemination volume among core users (coefficient 4.306, *p* < 0.01), but have a negative effect on intermediate users (coefficient −0.344, *p* < 0.05). In contrast, negative sentiment significantly increase dissemination volume among peripheral users (coefficient 0.383, *p* < 0.01), suggesting that negative content is more likely to trigger dissemination behavior among peripheral users.

Other factors, such as the number of followers and gender, also have a significant influence on dissemination volume. The number of followers has a significant positive effect on the dissemination volume across all user groups, with the strongest impact on peripheral users (coefficient 1.990, *p* < 0.01). Gender also plays a significant role in the dissemination volume of both core and peripheral users, with male core users exhibiting significantly higher dissemination volumes than their female counterparts (coefficient 0.513, *p* < 0.05).

In summary, the key role of core users in information diffusion is driven by topic relevance, forwarding enthusiasm, and positive sentiment. The dissemination behavior of intermediate users is more reliant on forwarding depth, while peripheral users are more sensitive to negative sentiment and topic relevance.

To verify the robustness of the negative binomial regression model, an additional analysis was conducted using linear regression (OLS). The dissemination data were log-transformed to meet the assumptions of the linear regression model. The results show that the core variables are highly consistent across both models, indicating that the OLS regression results align with the conclusions drawn from the negative binomial regression. This further validates the robustness of the model and provides reliable support for the study's conclusions.

## 5 Discussion

### 5.1 Main findings

This study is structured around four key research questions and employs multi-layered dissemination network modeling, topic identification, dynamic analysis of user roles, and regression models to derive the following key findings:

1) Public opinion dissemination on social media follows a multi-layered dissemination network model.

The study constructs a multi-layered dissemination network, where users are represented as nodes and reposting relationships as edges. The degree distribution of the network follows a power-law distribution, exhibiting typical social network characteristics. Dynamic network analysis further reveals the layered structure and temporal evolution patterns of public opinion dissemination, demonstrating significant differences in dissemination pathways and dissemination speeds across different circles. This finding validates the general applicability of the multi-layered dissemination network model in public opinion events, offering a novel perspective for understanding the structural characteristics of complex public opinion dissemination.

2) The multi-layered dissemination network consists of distinct topic circles.

Using the LDA model, the study identifies six primary topic circles, including “Product Quality and Hormone Controversy” and “Parental Concerns about Eczema Treatment.” Each topic circle exhibits distinct characteristics in terms of dissemination breadth, depth, and speed. For instance, the “Product Quality and Hormone Controversy” topic experienced the highest level of engagement in the early stages of dissemination, whereas the “Corporate Responsibility and Regulation” topic gradually became the focus in the later stages. These results suggest that the multi-layered dissemination network is composed of multiple independent yet interconnected topic circles, with their evolution reflecting dynamic shifts in public concerns.

3) User roles within topic circles significantly influence dissemination.

Users are classified into three distinct groups: core users, intermediate users, and peripheral users. Core users, despite being the fewest in number, exhibit the highest dissemination capacity, driven by topic relevance, forwarding enthusiasm, and positive sentiment. Intermediate users, characterized by their high mobility, function as information bridges. Peripheral users, while the most numerous, have limited influence on dissemination and are more driven by negative sentiment and topic relevance. Role transition matrix analysis further reveals that core users maintain the highest role stability, whereas intermediate users demonstrate greater mobility, with the potential to either ascend to core users or revert to peripheral users during the dissemination process. These findings highlight the dynamic function of user roles in public opinion dissemination, offering theoretical insights into information diffusion mechanisms.

4) The effectiveness of multi-layered public opinion dissemination is driven by multiple factors.

Through a negative binomial regression model, the study finds that topic relevance, forwarding enthusiasm, forwarding depth, and sentiment are key determinants of dissemination effectiveness. Topic relevance exerts a significant positive impact on dissemination across all user groups, with the strongest effect observed among peripheral users. Forwarding enthusiasm and forwarding depth significantly enhance dissemination among core users, suggesting that their rapid engagement and wide diffusion play a crucial role in maximizing dissemination outcomes. Sentiment exerts varying influences across user groups: positive sentiment significantly fosters dissemination among core users, whereas negative sentiment is more influential among peripheral users. These findings suggest that the effectiveness of public opinion dissemination is shaped by an interplay of user roles, topic relevance, sentiment, and forwarding depth, with different user groups exhibiting varying sensitivities to these factors.

### 5.2 Theoretical contributions

Firstly, this study reveals the multi-dimensional characteristics of topic circles and the dynamic patterns of their popularity changes, addressing the limitations of existing research on static topic dissemination. It expands the understanding of public opinion dissemination dynamics, with a particular emphasis on the temporal evolution of topic popularity and its features across different stages of dissemination. This provides a novel perspective for studying complex public opinion dissemination processes.

Secondly, this study is the first to explore the dynamic role transitions of core users, intermediate users, and peripheral users within public opinion dissemination networks. It addresses the gap in existing research on user classification (10), particularly in relation to the dynamic nature of user roles. Notably, it unveils the role of intermediate users as information bridges within networks, offering a new theoretical perspective on the mechanisms of role interaction and their dynamic behavior. Furthermore, this study extends the traditional static user classification framework, providing theoretical and methodological contributions to the analysis of structural characteristics and role transitions in dynamic and complex network dissemination.

Thirdly, this study uncovers the layered effects of emotional dissemination in public opinion networks, demonstrating that positive sentiment primarily drive the dissemination behaviors of core users, while negative sentiment significantly propel the behaviors of peripheral users. This layered effect complements existing research on sentiment dissemination (4, 14), which has primarily focused on the overall impact of sentiment. It highlights the heterogeneous roles of sentiment across different user groups and addresses the gap in existing theories regarding the interaction between user roles and sentiment dissemination.

### 5.3 Practical implications

This study offers significant practical implications for public health crisis management and public opinion monitoring, specifically in the following areas:

First, it provides guidance for public opinion management and crisis response. By uncovering the amplification effects of core users in information dissemination, this study highlights the importance of prioritizing the identification of core users. Crisis managers can strategically disseminate authoritative information and elicit positive sentiment to enhance the leadership role of core users in public opinion dissemination. Such strategies enable the rapid stabilization of public sentiment and effectively prevent the spread of negative public opinion.

Second, this study supports the dynamic monitoring of public opinion risks and dissemination nodes. By analyzing the evolving roles of users, it provides a scientific foundation for real-time public opinion monitoring. The role transition matrix reveals potential changes among intermediate users, underscoring the need for managers to track their role dynamics closely. Early identification of risks associated with intermediate users transitioning into core users allows for timely and precise interventions to mitigate the intensity of negative public opinion dissemination.

The findings contribute to the optimisation of information dissemination strategies. Sentiment dissemination research indicates that crisis management efforts should focus on disseminating positive and constructive information to core users to enhance the reach of positive public opinion. Simultaneously, timely clarifications or reassuring information should be issued to peripheral users to reduce the diffusion of negative emotions and minimize the adverse impacts of crisis-driven public opinion.

Furthermore, the findings of this public opinion analysis not only reveal public concerns regarding the quality of infant care products and hormone-related issues but also provide valuable informational support, assisting individuals in making more informed public health and medical decisions. Specifically, the following aspects illustrate how public opinion analysis findings can be utilized:

- Identifying high-risk products: By analyzing public discussions on Topic 1, high-risk products (e.g., antibacterial creams containing hormones) can be quickly identified, enabling consumers to avoid purchasing or using potentially hazardous products, thereby safeguarding infant health.
- Understanding the benefits and risks of treatment options: In Topic 2, the public expressed concerns about the use of hormone-based ointments. Public opinion analysis enables individuals to gain a more comprehensive understanding of the advantages and disadvantages of hormone treatments, allowing them to make more informed treatment decisions under medical guidance.
- Enhancing consumer awareness of rights: In Topic 5, the public raised concerns regarding corporate responsibility and regulatory deficiencies. These discussions can heighten consumer awareness of their rights, encourage greater public scrutiny of product quality and safety, and drive regulatory authorities to strengthen oversight.
- Promoting scientific communication: Public opinion analysis findings can assist scientific communication institutions and media in better understanding public concerns and uncertainties, thereby enabling more targeted science dissemination efforts that help the public accurately comprehend complex issues such as hormone treatments and product quality.
- Supporting policy formulation: Government agencies can utilize public opinion analysis findings to understand public concerns and demands regarding infant care products, thereby formulating more scientifically grounded and effective regulatory policies to safeguard public health.

Finally, this study provides valuable insights for public health decision-making. By analyzing public opinion posts, individuals can identify high-quality information sources, assess the impact of sentiment on decision-making, leverage dynamic dissemination patterns to optimize choices, and enhance health awareness through active participation in public discourse.

### 5.4 Limitations and future research

This study has certain limitations. Firstly, the data source is restricted to Sina Weibo, which may not comprehensively reflect user behavior across other platforms. Future research could incorporate data from multiple platforms to enhance the generalisability of the findings. Secondly, the classification of sentiment is relatively coarse. Future studies could leverage deep learning techniques to analyse more fine-grained emotions (e.g., anger, fear) to more accurately reveal the role of sentiment in public opinion dissemination. Moreover, the representativeness and potential biases of social media data should be further examined in future research, particularly concerning differences in participation and expression across various demographic groups on social platforms. Future studies should also explore the impact of different types of messages on public health decision-making, particularly the relationship between sentiment dissemination and health behaviors, to assist the public in making more informed choices during health crises. Finally, the causal inference in this study has not yet been fully validated. Future research could integrate experimental designs or time series analysis to enhance the reliability of causal inference.

## Data Availability

The raw data supporting the conclusions of this article will be made available by the authors, without undue reservation.
